# Magnetic Resonance Imaging‐Compatible Optically Powered Miniature Wireless Modular Lorentz Force Actuators

**DOI:** 10.1002/advs.202002948

**Published:** 2020-12-04

**Authors:** Senol Mutlu, Oncay Yasa, Onder Erin, Metin Sitti

**Affiliations:** ^1^ Physical Intelligence Department Max Planck Institute for Intelligent Systems Stuttgart 70569 Germany; ^2^ Department of Electrical and Electronics Engineering Bogazici University Istanbul 34342 Turkey; ^3^ Carnegie Mellon University Mechanical Engineering Department Pittsburgh PA 15213 USA; ^4^ School of Medicine and School of Engineering Koc University Istanbul 34450 Turkey; ^5^ Institute for Biomedical Engineering ETH Zurich Zurich 8092 Switzerland

**Keywords:** Lorentz force actuator, magnetic resonance imaging, MRI‐compatible, optical actuation, wireless actuation

## Abstract

Minimally invasive medical procedures under magnetic resonance imaging (MRI) guidance have significant clinical promise. However, this potential has not been fully realized yet due to challenges regarding MRI compatibility and miniaturization of active and precise positioning systems inside MRI scanners, i.e., restrictions on ferromagnetic materials and long conductive cables and limited space around the patient for additional instrumentation. Lorentz force‐based electromagnetic actuators can overcome these challenges with the help of very high, axial, and uniform magnetic fields (3–7 Tesla) of the scanners. Here, a miniature, MRI‐compatible, and optically powered wireless Lorentz force actuator module consisting of a solar cell and a coil with a small volume of 2.5 × 2.5 × 3.0 mm^3^ is proposed. Many of such actuator modules can be used to create various wireless active structures for future interventional MRI applications, such as positioning needles, markers, or other medical tools on the skin of a patient. As proof‐of‐concept prototypes toward such applications, a single actuator module that bends a flexible beam, four modules that rotate around an axis, and six modules that roll as a sphere are demonstrated inside a 7 Tesla preclinical MRI scanner.

## Graphical abstract

Wireless, optically powered, miniature, modular, and magnetic resonance imaging (MRI) compatible Lorentz force actuators are proposed. Single actuator bending a flexible beam, four actuators forming a rotor, and six actuators forming a rolling sphere have been demonstrated inside a 7 Tesla MRI scanner. They can be used in future interventional MRI applications, e.g., positioning biopsy needles on patient's skin.

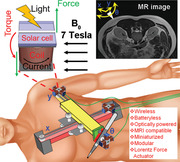

## 1 Introduction

Interventions inside magnetic resonance imaging (MRI) scanners have increasingly gained interest due to the need for high‐resolution, three‐dimensional (3D), and nonionizing soft tissue imaging during complex medical procedures. Interventional MRI (iMRI), as a newly emerging field, helps guide minimally invasive medical operations not only with its imaging feedback but also with in vivo temperature, diffusion, ventilation, and perfusion 3D imaging capabilities.^[^ ^1^ ^]^ Such field paved the way for the development of MRI‐compatible robotic actuation systems in an effort to overcome limited access of the confined in‐bore space of an MRI scanner and to further improve the placement accuracy of interventional medical tools.^[^ ^2^ ^]^

MRI scanner presents a challenging environment for active devices inside with its high, axial, and uniform magnetic field (***B*_0_**), fast‐switching magnetic field gradients, and radio frequency (RF) pulses.^[^ ^3^ ^]^ Ferrous objects can be harmful projectiles under the influence of high magnetic forces exerted by high magnetic field gradients inside and outside of the scanner. They also create large magnetic resonance (MR) imaging artifacts.^[^ ^4^ ^]^ RF pulses and magnetic field gradient coils can heat long conductive wires, causing injury or discomfort to the patient.^[^ ^5^, ^6^ ^]^ Its imaging system is also very sensitive to external electromagnetic interference, such as created by current‐carrying wires that pass from the outside to the inside of the scanner room. Hence, MRI compatibility of actuation systems is very crucial for the iMRI field. A device is considered MRI‐compatible when it does not present any additional risk to the patient and the scanner and its presence does not significantly affect the quality of the MR images.^[^ ^3^ ^]^

Extensive research has been conducted on MRI‐compatible hydraulic, pneumatic, and ultrasonic tethered actuators for interventions, such as brain biopsies, breast interventions, endoscope manipulation, and prostate, liver, kidney, and general percutaneous procedures.^[^ ^7^ ^]^ Hydraulic actuators suffer from cavitation and fluidic leakage. Pneumatic actuation is back‐drivable and difficult to control at the millimeter scale because of the compressibility of air and the induced time delay. Even though ultrasonic ones are mostly favored, they employ high‐frequency electrical signals.^[^ ^8^ ^]^ They are considered MR‐compatible only when they are away from the imaging area. With their motor staying in a distance, a motion transmission system composed of linkages, driveshafts, belts, chains, etc. is used resulting in joint flexibility, backlash, and friction problems.^[^ ^3^ ^]^ Remote actuation is usually associated with low bandwidth and resolution owing to their bulky nature. Tethered solutions are challenging to miniaturize to mitigate limited in‐bore space problem of MRI scanners.

Therefore, recent studies have started to focus on tetherless robotic systems with a goal of further miniaturization. On‐site actuation is realized by utilizing the inherently available constant high magnetic field ***B*_0_** or precisely programmable 3D magnetic field gradients of MRI scanners. Navigation and steering have been demonstrated by the generation of magnetic pulling forces on ferrous beads using the 3D magnetic gradient coils of the scanners.^[^ ^4^, ^9^, ^10^, ^11^, ^12^ ^]^ However, these methods require additional modifications at the software and imaging sequence levels. More importantly, because each ferrous object creates a large distortion (at least ten times of its size) on MR images, which cannot be turned off, it is not possible to acquire the surrounding tissue MR images. Their actuation force is proportional to the volume of the particle and magnitude of the magnetic field gradients, which makes the force very small when the particle size is scaled down to a few millimeter size scale. Also, conventional clinical MRI scanners have a limited maximum magnetic gradient of 40 mT m^−1^. Such limited gradients and particle volume scaling severely put a limit on the miniaturization of such actuators.^[^ ^10^ ^]^

Lorentz force‐ or torque‐based actuation methods, on the other hand, are more promising for iMRI applications owing to the large ***B*_0_** (e.g., 3–7 T) inside MRI scanners. Such a strong magnetic field allows the exertion of larger Lorentz forces compared to the magnetic pulling forces at smaller scales.^[^ ^5^ ^]^ Promising results of wired Lorentz force actuators have been reported for beam bending and catheter guiding.^[^ ^13^, ^14^, ^15^, ^16^ ^]^ However, long electrical power lines pose hazards inside the MRI scanner. Moreover, the employment of batteries is not practical since their size and weight cannot be made smaller and they have limited lifetime.^[^ ^17^ ^]^ In this perspective, wireless power transmission (WPT) stands out as a viable solution. Major WPT and energy harvesting technologies involve inductive coupling,^[^ ^18^, ^19^, ^20^ ^]^ ultrasonic,^[^ ^21^ ^]^ photovoltaic,^[^ ^22^, ^23^, ^24^ ^]^ electrostatic,^[^ ^25^ ^]^ electromagnetic,^[^ ^26^ ^]^ thermoelectric,^[^ ^27^ ^]^ and triboelectric^[^ ^28^ ^]^ methods. Among them, photovoltaic technologies deliver the greatest amount of power in the smallest volume.^[^ ^29^ ^]^ Furthermore, optical WPT is MRI compatible and does not cause any electromagnetic interference problems.^[^ ^30^ ^]^ Optical powering has been utilized in implantable integrated microsystems to monitor intraocular pressures,^[^ ^31^ ^]^ neural recorders,^[^ ^32^ ^]^ retinal prosthesis,^[^ ^33^ ^]^ and pacemakers.^[^ ^34^ ^]^ Although utilization of WPT has critical importance for iMRI applications, very few studies have been conducted in this direction so far.^[^ ^35^ ^]^

**Figure 1:**
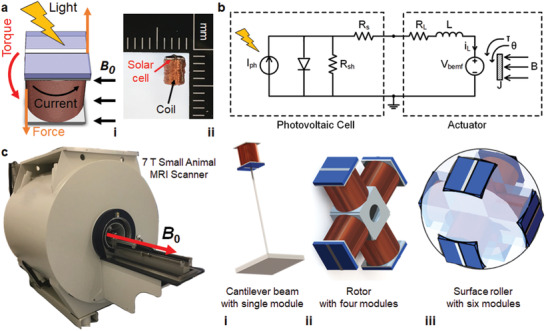
Optically driven miniature wireless Lorentz force actuator design inside an MRI scanner with single‐ and multimodule actuation demonstrations. a,i) Schematic description of a single actuator module with the related physical parameters involved and ii) photograph of an experimental actuator module prototype with its components. b) Equivalent electrical circuit diagram of the actuator module. c) Building different actuation mechanisms inside a preclinical MRI scanner (***B*_0_** = 7 T) using different number of actuator modules: i) one wireless module that can bend a flexible cantilever beam, ii) four wireless modules that can rotate around an axis and iii) six wireless modules inside a spherical shell that can rotate around itself and roll on surfaces along ***B*_0_** direction.

In this work, we introduce an MRI‐compatible, modular, and wireless Lorentz force actuator powered optically. The actuator consists of a photovoltaic cell and a coil. Lorentz force/torque is generated on the coil under high ***B*_0_** of the MRI scanner with the electrical current generated by the received optical power transmission as depicted in **Figure** **1**a. Wireless nature and high ***B*_0_** enable miniaturization of the actuator module down to a few millimeter scale and optical power transfer enables the MRI compatibility. Instead of designing a specific MRI‐compatible actuator or robotic system, we develop a modular wireless Lorentz actuator and then integrate multiple of such modules to show proof‐of‐concept demonstrations that can be used in future iMRI applications. Conceptual drawings of the demonstrations implemented in this work along with the image of the used preclinical 7 T MRI scanner are shown in Figure 1c. A single module attached to the tip of a cantilever beam bends it inside the MRI scanner. Four modules are integrated together to form a rotor. Six modules are used to form a spinning and rolling wireless sphere.

## 2 Results

### 2.1 Components of the Wireless Actuator Module and Its Actuation Principle

Wireless Lorentz force actuator primarily consists of two components: a photovoltaic cell, which can be a solar cell, photodiode, or a light‐emitting diode (LED), and a coil. The working principle of the actuator is according to the Lorentz law. Moving electrons on a current‐carrying wire under a magnetic field experience a force perpendicular to both the direction of the current and the magnetic field such that

(1) $$\begin{array}{c} {\vec{F}}_{\mathrm{Lorentz}}=\;I\int d\vec{l}\times \vec{B} \end{array}$$

where *I* is the flowing current, $d\vec{l}$ is the infinitesimal wire length vector, and $\vec{B}$ is the external magnetic field vector present on the wire. For a closed loop of wire, there cannot be any net force, however, a net torque is induced on the loop. For a coil with multiwindings, the Lorentz torque vector can be represented as

(2) $$\begin{array}{c} {\vec{\tau }}_{\mathrm{Lorentz}}=\;IN\left(\vec{A}\times \vec{B}\right) \end{array}$$

where *N* is the total number of windings and $\vec{A}$ is the area plane vector of the loops. In the presented work, the current to the module (*I*) is supplied wirelessly by optical power through a solar cell (Figure 1a,i).

The significant challenge in the design and fabrication of such actuator is to make all the components nonferromagnetic in order to achieve MRI compatibility. Even though most of the unpackaged electronic components, such as integrated circuits, transistors, diodes, and LEDs, do not contain any magnetic material, almost all the available off‐the‐shelf electronic components are packaged and their packages contain magnetic materials. Therefore, as a photovoltaic cell of our actuator, an unpackaged monocrystalline silicon solar cell is diced into smaller dies of 2.0 × 2.4 mm^2^. Coils are also wound in‐house using copper wires of 50 µm diameter (Figure 1a,ii). The resulting actuator module dimensions are 2.5 × 2.5 × 3.0 mm^3^. This small‐sized module prototype is expected to generate maximum force and torque values of 96 mN and 197 μNm, respectively with 10 mA current through its coil under optical power of 7 mW mm^−2^, where ***B*_0_** = 7 T (see Note S1, Supporting Information). An optically powered actuation proof‐of‐concept demonstration of free wireless actuator modules on the surface of a 0.1 T permanent magnet by a spot of laser beam light can be found in Movie S1 in the Supporting Information.

### 2.2 Circuit Model of the Module

**Figure 2:**
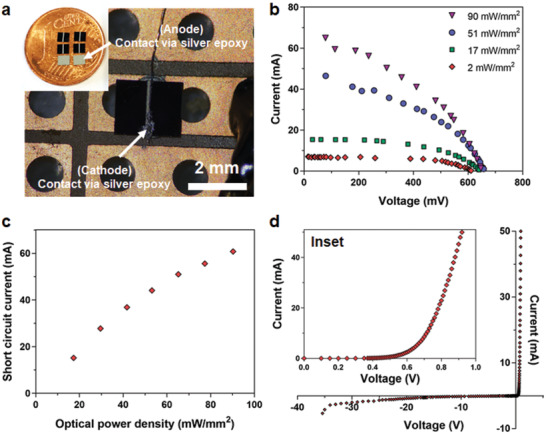
Characterization results of the solar cell die. a) Photograph of diced solar cells on a penny and its connection to a printed circuit board for characterization. b) Current–voltage characteristic of the solar cell under different 808 nm laser illumination conditions. c) Short‐circuit current generated by the solar cell under different 808 nm laser illuminations. d) Current–voltage characteristics of the solar cell under dark for reverse and forward biased conditions. The inset shows close view of the forward‐biased region.

Developing the electrical circuit model of the actuator is important in order to analyze and estimate the generated forces and torques and to optimize its power efficiency. The circuit model of the actuator (Figure 1b) consists of a solar cell equivalent circuit model and the circuit model of the coil. The equivalent circuit model of the solar cell consists of an ideal current source, photogenerated current, which has a value proportional to the light power intensity. The diode in the circuit models the p–n junction behavior of the solar cell. The circuit also has series and shunt resistors to model the nonidealities of the solar cell's energy harvesting capability. These parameters are extracted from the solar cell measurements in **Figure** **2**. The coil is modeled as an inductor and its series resistance. Since this coil is moving inside a magnetic field, voltage is generated across its terminals opposing this motion. This generated voltage (*V*_bemf_) is known as the back electromotive force (EMF). It is modeled as an ideal voltage source shown in Figure 1b. For a constant magnetic field, its value is found from

(3) $$\begin{array}{c} V_{\mathrm{bemf}}=-N\frac{d\left(\vec{B}.\vec{A}\right)}{dt} \end{array}$$

### 2.3 Characterization of the Photovoltaic Cell

Monocrystalline silicon solar cells can harvest energy from a wide spectrum of light ranging from 400 to 1100 nm wavelengths, with peak sensitivity in the near‐infrared (IR) region of around 780–980 nm.^[^ ^36^ ^]^ Hence, WPT to the solar cells can be achieved with different light sources and wavelengths. In this work, 785 and 808 nm laser light sources, a halogen lamp, and 850 and 940 nm power‐LEDs are used for WPT. For the characterization of the solar cells, 2.0 × 2.4 mm^2^ solar cells are placed on a printed circuit board (PCB) and their anode and cathode connections are made via silver epoxy bonding (Figure 2a). Current–voltage characteristics of the solar cells are measured under different light power densities of different light sources, i.e., 2 mW mm^−2^ of 785 nm laser and from 2 to 90 mW mm^−2^ of 808 nm laser. The resulting current–voltage graph in Figure 2b shows the generated electrical power for the given light intensities. The slopes of these curves are used to extract series, *R*_s,_ and shunt, *R*_sh_, resistance values in the circuit model of the solar cell (Figure 1b). The solar cell's biasing current and voltage values for a given optical power density would be at a point on these graphs depending on the impedance of the load connected to the solar cell, i.e., the resistance and inductance of our actuator. The short‐circuit response of the solar cells to different optical power densities is shown in Figure 2c. They are used to find photogenerated current source values, *I*_ph_. These values change linearly with the applied optical power densities.However, saturation starts for densities above 50 mW mm^−2^.

Forward‐ and reverse‐biased characterizations of solar cells are performed under dark conditions to determine the electrical model parameters of the diode in Figure 1b. The forward bias characteristic of the solar cell is a typical silicon diode behavior with a turn‐on voltage of around 0.65 V (Figure 2d and its inset). Small current values on the order of μA pass for several volts of reverse bias voltages. The reverse breakdown starts to happen at around −20 V with a small leakage current of around 0.8 mA. This increases to 2 mA at −30 V. High reverse currents around 5 mA passes only at voltages of −35 V and above (Figure 2d). This low current leakages of the solar cell at elevated reverse voltages are important as it will be evident in the discussion of the rotor operation inside the MRI in the context of back EMF generation.

### 2.4 Single Wireless Actuator Module Characterization in a Cantilever Beam Bending Setup

**Figure 3:**
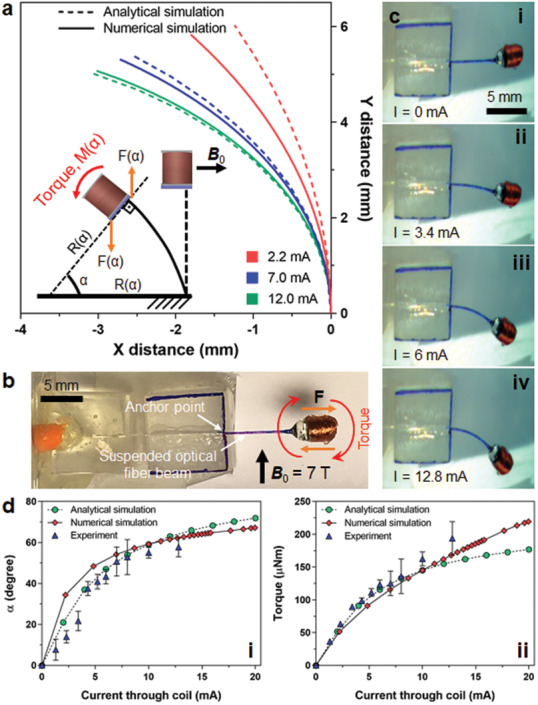
Simulation and experimental test results of the single wireless module attached to a cantilever beam. a) Analytical and numerical simulation results showing the bending profiles of the actuator attached to the tip of an optical fiber with different electrical current values passing through its coil under the uniform magnetic field of 7 T. b) Photograph of a single module characterization setup using 7 mm long optical fiber (105 µm diameter silica core) as suspended cantilever beam, which makes sure that delivered optical power stays almost constant with bending. c) Experimental bending profiles of the actuator for different laser light intensity values resulting in the mentioned current values passing through its coil inside preclinical MRI scanner (***B*_0_** = 7 T). d) Analytical and numerical simulation and experimental results inside preclinical MRI scanner (***B*_0_** = 7 T) showing the saturation of the beam bending angle with different current values passing through the coil and the corresponding torque values under the same condition. The error bars represent the standard error of the mean (*n* = 3).

A single actuator module can be utilized at the tip of a cantilever beam to bend it as an actuation example. Useful iMRI applications require bending actions as in marking a needle insertion point or adjusting the slope of its insertion. A simple bending analysis is developed analytically to relate beam dimensions and applied currents to the generated bending angles and torques. Depiction of this beam bending, related physical quantities, and the bending profile results of this analysis are plotted in **Figure** **3**a along with its comparison to numerical solutions of the finite element analysis. In the analytical and numerical analysis, the actuator is attached to an optical fiber with a length of 7 mm and a silica core diameter of 105 µm. This way, the optical fiber is used as a cantilever beam. This makes the actuation more efficient since the light power to the solar cell stays almost constant as the beam bends. Initially, the actuator is placed orthogonal to ***B*_0_**, *α* bending angle being 0°, which is the point of maximum torque generation. As optical power is inserted into the fiber, it generates an electrical current in the coil by the solar cell. The generated torque actuates the coil trying to align its magnetic moment vector with ***B*_0_**. As the coil bends, the bending angle increases, which in turn reduces the generated torque. Hence, it gets harder to achieve higher bending angles.

In the bending analysis, it is assumed that the beam bending profile is symmetrical in the form of a circular arc. Using a cylindrical fiber with a radius of *r*, the following relation can be derived from beam bending analysis in response to an externally applied moment/torque^[^ ^13^, ^14^, ^16^, ^37^ ^]^

(4) $$\begin{array}{c} \frac{\alpha }{\cos\alpha }=\frac{4BINAL}{E\pi r^{4}}\; \end{array}$$

where *α*, *E*, and *L* are the bending angle, Young's modulus, and the length of the beam, respectively. The derivation details of this analytical equation are given in Note S2 in the Supporting Information. The agreement between analytical and numerical solutions get closer as the applied current through the coil is increased from 2.2 to 12 mA. The mismatch between analytical and numerical results up to that point is due to the assumption of circular bending profile made by the analytical analysis.

For experimental measurements, laser light of different intensities at 808 nm wavelength is applied to the actuator that is attached to the tip of an optical fiber. The fiber is butt‐coupled to the solar cell of the module using an epoxy. The fiber is anchored to the plastic blocks of a container so that 7 mm length of the fiber is suspended in air (Figure 3b). The parameters of the optical fiber are the same as the ones used in the analytical and numerical analysis. The bending of the cantilever beam is demonstrated inside the 7 T MRI scanner (Movie S2, Supporting Information). After acquiring different bending angles for different laser settings, the current through the coil is measured outside the MRI scanner for the same laser power settings. These values change from 0 to 12.8 mA. Sample photographs of bending are included in Figure 3c for the mentioned coil current values. The change of the bending angle and torque values for these current values of the coil is plotted in Figure 3d for analytical, numerical, and experimental results. Current value of 10 mA passing through the coil causes 55° bending on the beam. Both the analytic and numerical results estimate this to be 59°. This bending results in 163 μNm torque value at its static equilibrium. This means a torque value of 284 μNm is achievable at a bending angle of zero using the same current value. These results suggest that it becomes inefficient to bend the beam at degrees above 55° since it requires more current to get small improvements in the bending angle after this point. This indicates the necessity of using a second module orthogonal to this one for more complex operations as commonly done in the wired Lorentz force actuator designs.^[^ ^14^, ^15^, ^16^ ^]^

### 2.5 MRI Compatibility of the Actuator Module

**Figure 4:**
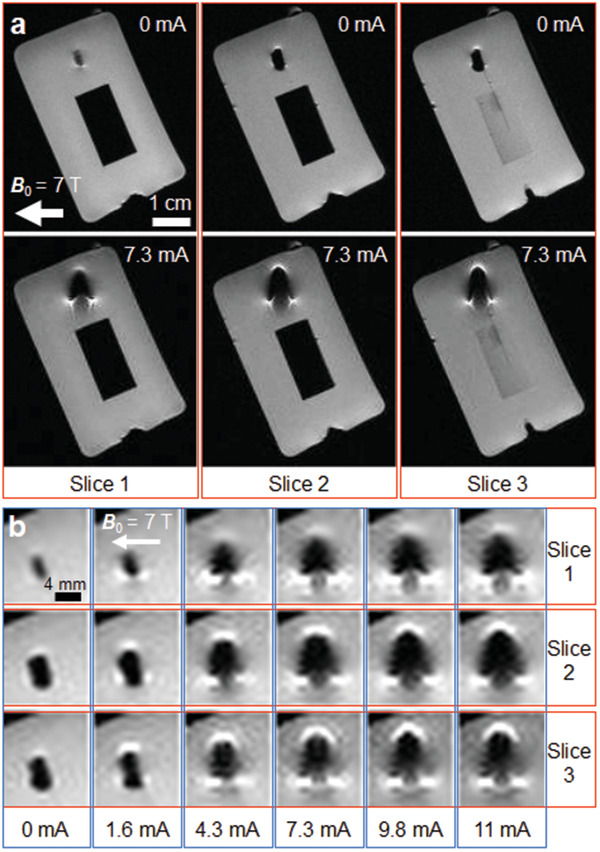
MR image artifact characterization of the single wireless actuator module in off and on states. a) Three different MR image slices of the nonoperated (off, 0 mA) actuator showing no image artifact and operated actuator showing beam bending and generated image artifacts inside preclinical MRI scanner (***B*_0_** = 7 T). b) Growing image artifacts are shown at three different image slices of the coil with increasing applied current passing through it by the application of different laser intensities.

For iMRI usage, it must be shown that when the actuator is not operated, it does not cause any artifact in the MR images around the actuator. The results of these tests can be seen in **Figure** **4**. The same actuator attached to the optical fiber is inserted to the MRI scanner, now inside a phosphate‐buffered saline (PBS) solution. Water content is necessary for MRI in order to generate an imaging signal. In the absence of hydrogen proton excitation and precession, the images would appear all black similar to the black appearance of plastic objects in MRI. The usage of PBS also helps emulate the physiological imaging environment. Different imaging sequences are applied to acquire the MR images of actuators that use different 808 nm laser power settings. Figure 4a images are taken using rapid acquisition with relaxation enhancement (RARE) MR imaging sequence protocol. Three different slices of the coil and its surroundings are shown in the acquired MR images. When the actuator is not operated there is no artifact in the images. However, when laser power is set to result in 7.3 mA current in the coil, the bending of the beam in water solution and artifact generation around the coil is visible. This is expected because a current‐carrying coil generates its own magnetic field and this distorts ***B*_0_** locally, which results in artifacts in the image. Using a faster but four times lower resolution image sequence protocol, fast spin‐echo for short echo time using slew‐rate‐optimized gradients (RAREst), a detailed characterization of the artifact growth is made for different laser power settings as shown in Figure 4b. Again, three different slice images of the coil are shown as the coil current is swept from 0 to 11 mA. When the actuator is off, there is no image artifact. As current is applied and increased, the growth of the artifact is visible in every slice. The growth saturates at current values above 7.3 mA. When operated, the maximum size of the artifact is around two times the size of the module, which is still much less than the size of the artifacts created by ferrous particles (typically at least ten times of the particle size). Observable small artifacts are sometimes formed intentionally in iMRI by passing small electrical currents through microcoils to actively visualize and track catheter tips.^[^ ^38^ ^]^ So, such small MR image artifacts can be used as a high‐contrast medical tool tracking in the future also, while not affecting the physiological MR image distortion around the tool too much.

### 2.6 Actuation of the Rotor Built with Four Wireless Actuator Modules

**Figure 5:**
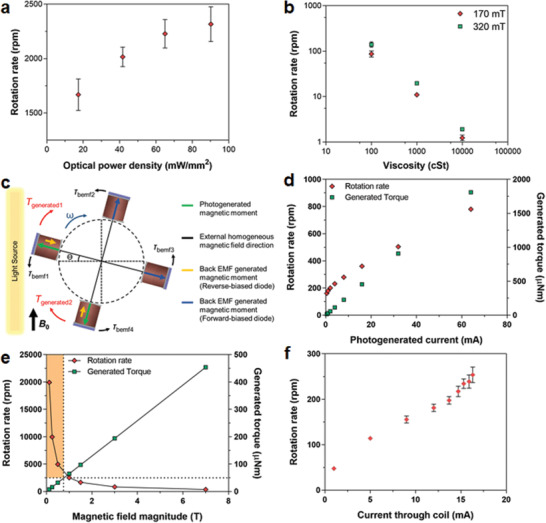
Characterization results of the wireless four‐module rotary actuator. a) Experimental measurements of the rotation rate of the rotor in the air under different optical intensities on a permanent magnet of 0.1 T. b) Experimental measurements of the rotation rate of the rotor in silicone oil of different viscosities on magnets with different magnetic field values under the optical light intensity of 12 mW mm^−2^. c) Modeling of the rotor inside a uniform magnetic field. d) The HSPICE simulation results of the rotor inside the uniform magnetic field of 7 T showing rotation rates and generated torque values for different photogenerated currents of the solar cells. e) The HSPICE simulation results of the rotor inside the uniform magnetic field of different values for 16 mA photogenerated current of the solar cell showing rotation rates and generated torque values. f) Experimental measurements of the rotation rate of the rotor in the air for different laser intensities resulting in different current values through the coils inside preclinical MRI scanner (***B*_0_** = 7 T). In (a), (b), and (f), the error bars represent the standard error of the mean (*n* = 3).

The rotor is built simply by adhering four of the actuator modules orthogonally to each other around a 2 mm sized cubic plastic block that is 3D printed. The center of this block is hollow along the rotation axis so that any string or a rod can be attached during tests. The functionality of the rotor is first tried on the surfaces of permanent magnets in air. High rotation rates on the order of 1000 rotation‐per‐minute (rpm) are achieved using 0.1 T as shown in **Figure** **5**a. The maximum rotation rate of 2300 rpm is measured for an optical intensity of 90 mW mm^−2^ of 808 nm laser. Rotation rates are measured using a high‐speed camera at 1000 frames per second (fps). The measurements show close to a linear relationship with optical power intensity while saturation starts for values above 50 mW mm^−2^. The reason for this is the saturation of the solar cell's photogeneration current at these high optical powers. This saturation was characterized earlier in the results of the short‐circuit current in Figure 2c. For the given optical power density, photogenerated current values can be calculated from the short‐circuit characterization results of Figure 2c. Using the circuit model of the actuator, the current that must pass through the coil in response to these optical power densities can be predicted. When the current values that pass through the coils are plotted against rotation rates, then the results show a linear relationship without saturation. Movie S3 in the Supporting Information shows the operation of a rotor on a 0.15 T magnet by a laser from a distance of 12 cm. Similar operation across 100 cm distance is also achieved. Longer working distances are possible as long as the dispersion on the collimated beam of the laser does not prevent delivering the required power to the solar cells.

Rotation of the optical rotor is also shown inside silicone oil over the surfaces of different permanent magnets. Three different viscosities of 100, 1000, and 10 000 cSt are tested. The reasons for these tests are to show that wireless optical rotors can generate high enough torques to work against higher viscous forces. The rotation rates are measured over 0.17 and 0.32 T magnets using a 20 W halogen lamp from a distance of 2 cm, giving approximately an optical power density of 12 mW mm^−2^ (Movie S4, Supporting Information). These results are summarized in Figure 5b.

### 2.7 Analysis of the Optical Rotor Inside a Uniform Magnetic Field

The magnetic field on permanent magnets is nonuniform with a rapidly decreasing magnitude away from its surface. This creates a gradient, which favors rotation because the modules not actuated by light are further away from the magnet surface, hence, they do not generate high back EMFs that oppose rotation. Inside MRI, on the other hand, there is a high ***B*_0_** field and this applies equally to all four modules. Therefore, a detailed analysis is required to understand the behavior of the optical rotor inside this uniform magnetic field. To simplify the complex analysis of a four‐module rotor operation, it is assumed that the rays of the light source are always straight and approach the rotor from one side covering its full diameter. This means that only two modules can generate electrical currents optically, namely module 1 and module 4 as depicted in Figure 5c. Assuming that all the modules rotate steadily with *ω*, the coil of every module generates back EMF with different polarities so that all of them oppose rotation, including the modules that generate the motion torques. Each module has a specific orientation with respect to the rotation angle, *θ*, and all of them are orthogonal to each other. Back EMF torques generated by these modules are labeled in Figure 5c. The generated back EMFs of modules 1 and 4 reverse‐bias their solar cells; whereas modules 2 and 3 forward‐bias. As characterized in Figure 2d, the reverse direction of the solar cell passes a very small amount of current even for tens of negative volts applied. As a result, very small back current can flow due to this back EMF and a negligible opposing torque is generated. The forward biased solar cells, on the other hand, can pass high currents resulting in large torque values opposing rotation. Detailed analytical and numerical analysis in Note S3 in the Supporting Information show that the rotation rate increases linearly with the current passing through the coils generated by light power and with the reciprocal of the magnitude of the uniform magnetic field.

Using extracted solar cell and coil circuit parameters, the mechanical response of the actuators are numerically solved with the current and voltage results generated by the Hailey simulation program with integrated circuit emphasis (HSPICE) computer‐aided design tool and related mechanical force and torque equations. Photogenerated and back EMF torques are calculated and transient results are plotted using this tool. Resulting rotation rate and torque values from these simulations are shown in Figure 5d, as the photogenerated current is changed from 0.5 to 64 mA for a fixed uniform magnetic field value of 7 T. In accordance with the analysis in Note S3 in the Supporting Information, there is a linear increase in rotation rates for increasing photogenerated currents. Similarly, simulation results of rotation rate and torque values are plotted in Figure 5e for a fixed photogenerated current of 16 mA as uniform magnetic field value is varied from 0.125 to 7 T. The results show the increase in rotation rate with the reciprocal of the magnetic field value. However, they also show one shortcoming of the simulations; very high rotation rates are calculated for almost zero magnetic field value. In reality, such high rotation rates at very low magnetic fields cannot occur because the rotor's initial start torque cannot be overcome. The static friction, the asymmetric center of mass and the gravity effects create resistance to rotation. Even if the rotor starts to move, drag forces, which increase with the square of the velocity of the rotor, would be comparable to the generated small torque at this low magnetic field and would slow and eventually stop the rotor. Therefore, the regions above 2500 rpm are shaded in Figure 5e. This overall analysis suggests slower rotation rates but higher torque values inside the high uniform magnetic field of the MRI scanner compared to the ones achieved using off‐the‐shelf permanent magnets.

### 2.8 MRI Scanner Test Results of the Four‐Module Rotor

**Figure 6:**
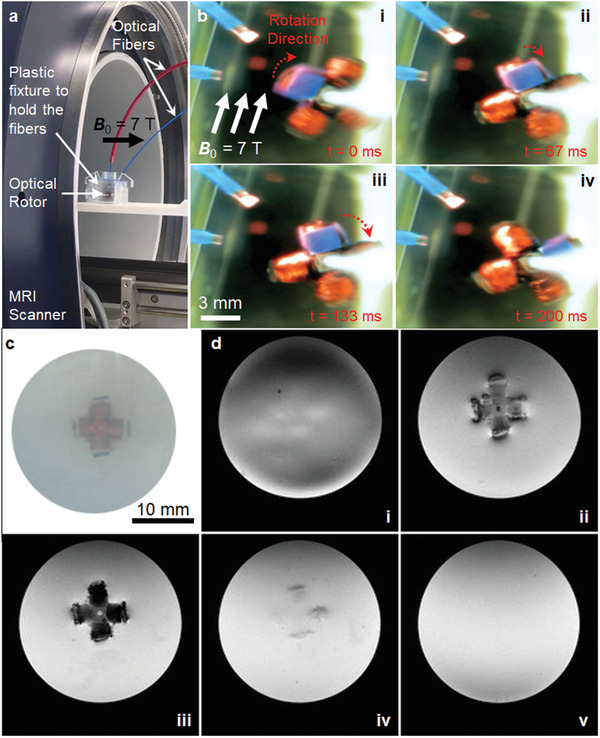
Test results of the four‐module rotary actuator inside the MRI scanner. a) Insertion of the rotor into the preclinical MRI scanner (***B*_0_** = 7 T) with the plastic holders and fiber optic cables. b) Representative time‐lapses of the rotor motion inside the MRI scanner with optical power applied through the fibers. c) Optical image of the rotor inside the agar gel. d) FLASH coronal MR image slices of the same rotor inside the MRI scanner showing no image artifacts when it is not operated (off).

A rotor is positioned inside a plastic box and two optical fibers are positioned around it with 45° between them. Insertion of the rotor into the MRI scanner with the plastic holder and fiber optic cables is shown in **Figure** **6**a. The orientation of the rotor inside the MRI is adjusted so that the fiber powers the actuator module that is orthogonal to ***B*_0_** = 7 T. This position generates the maximum initial torque. The second fiber that is 45° from it is used to self‐start the rotor. With a single fiber, it is very hard to start rotation because the fiber generates a small‐sized light spot that covers ≈45° of the perimeter of the rotor. The initial torque of the laser moves the rotor up to 45° but it cannot move further because the module now receives almost no light power. Also, the back EMFs of the other modules opposes this initial attempt to generate a continuous motion. The second fiber helps to pass this bottleneck. While the mentioned module makes this 45°, the light power emitted from the second fiber gives an extra push to it and also to its neighbor and the rotation starts. Representative time‐lapses of the rotor motion inside the MRI scanner with optical power applied through the optical fibers are shown in Figure 6b. The resulting average rotation rates are measured and plotted in Figure 5e for different current values that flow through the coils. These current values are extracted from the measurements of the solar cells that are made outside the MRI scanner using similar coils and the same optical power densities. As predicted by the simulation results discussed above, the rotation rates change almost linearly with the current values. These results can be viewed in Movie S5 in the Supporting Information as well. Detailed image analysis results of this movie show how rotation angle changes with time, which is plotted in Figure S3 in the Supporting Information for all coil current values. They show fluctuations in the rotation rates. Due to friction, the position of the center of the mass and gravity effects and also smaller light spots not covering the whole semi‐circle angles, the rotor slows down and speeds up. The simplified analysis discussed above does not include these nonidealities, hence, do not foresee these instantaneous rotation rate changes over a time period. These nonidealities also make the analysis overestimate the rotation rate values. Coil current of 16 mA in MRI tests results in an average rotation rate of 239 rpm whereas the HSPICE simulation for these parameters predicts 360 rpm. However, the developed analysis gives an intuition about the working mechanism of this type of rotor. It predicts correctly how rotation and torque values change with the change in the current and magnetic field values, e.g., slower rotation rates of MRI test results compared to the ones made on permanent magnets.

MRI compatibility is also tested for the rotor. It is placed inside an agar gel. Its MR images are acquired while not operated with the image sequence protocol of fast low angle shot (FLASH). The optical image of the rotor inside the agar gel is shown in Figure 6c. Its coronal MRI images in Figure 6d show no image artifacts. Its FLASH axial MRI images can be found in Figure S4 in the Supporting Information.

### 2.9 Actuation of a Spherical Roller Built with Six Wireless Actuator Modules

**Figure 7:**
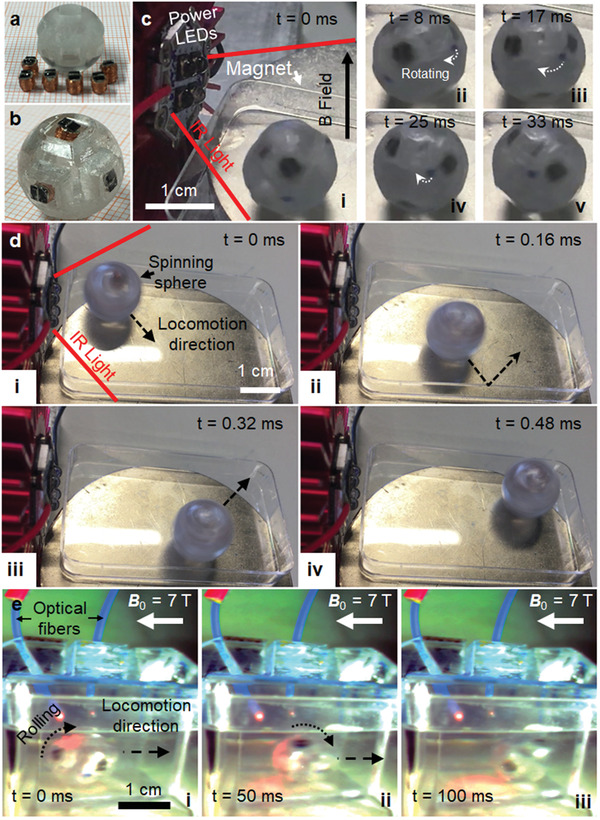
Characterization results of the wireless six‐module spherical actuator. a) Photograph of the 3D‐printed plastic sphere and six actuator modules before assembly. b) Photograph of the assembled spherical roller with six modules. c) Rotation of the spherical roller on a 0.25 T permanent magnet with the applied IR light at different time‐lapses. d) Motion of the spherical roller on a 0.25 T permanent magnet with IR light radiation as it spins and hits the walls of the plastic container. e) Rolling and forward motion of the sphere inside the preclinical MRI scanner (***B*_0_** = 7 T) inside a plastic container upon turning on IR light through two optical fibers.

A wireless rotating and rolling sphere has been built using six wireless actuator modules. A plastic sphere structure with six rectangular prism cavities is 3D printed as shown in **Figure** **7**a. The final device, shown in Figure 7b, is realized simply by placing and adhering six wireless modules into the cavities. This device can rotate around itself and roll on the surface along the external magnetic field direction. The initial actuation of this device is realized on a 0.27 T permanent magnet with a diameter of 6 cm. A 5.3 W power‐LED of 850 nm wavelength is placed on the side of the permanent magnet. The sphere starts to spin at rotation rates of around 1800 rpm by itself as soon as the IR LED light is turned on as shown in Figure 7c and Movie S6 in the Supporting Information. As it spins, it can also move forward, hit, and bounce from side walls as demonstrated in Figure 7d. The same sphere is also tested inside the MRI scanner. Similar to the rotor setup inside the MRI scanner, the sphere is positioned inside a plastic box and two optical fibers are positioned around it with 45° between them. Since solar cells are at discrete positions around the sphere, it is hard to actuate a specific solar cell using a single fiber. However, when two fibers send optical power, it can start the rotation of the sphere as discussed earlier. The initial torque given by the two fibers rolls the sphere and moves it forward along the ***B*_0_** direction as shown in Figure 7e and Movie S7 in the Supporting Information. Another example of rolling and forward motion of the sphere inside MRI scanner is demonstrated in Figure S5 in the Supporting Information. These results prove that such a wireless sphere could roll on surfaces, e.g., on the skin of a patient and position an MRI marker placed inside.

## 3 Discussions

Current iMRI procedures, such as biopsies, are often termed as in‐out. Patient is moved into the MRI for imaging and pulled out to insert a needle. This is time consuming and prevents precise positioning especially with moving patients and organs. The need to scan at the isocenter requires the physician to lean into the magnet bore while adjusting the interventional device under real‐time MR imaging manually in a very loud environment. For an efficient, safe, and effective procedure, it is important to continuously know where the interventional device is.^[^ ^1^ ^]^ The results in this work are important as they try to find solutions to these problems in the form of a remotely controlled robotic system by developing its building blocks. Physicians can use these kinds of actuation systems to mark the needle's entry point and orientation or position an MRI marker while evaluating the real‐time images of the patient comfortably in the operator room.

Applications of iMRI demand a small footprint for actuators. These applications also require sufficient force and torque to be able to bend a tip working as an MRI needle or move a marker on the skin of the patient. Even though optical WPT can deliver high power in small dimensions, there is a limit in sending wireless power by the healthcare safety regulations. For example, the maximum permissible exposure limit of skin for continuous operation of laser light is 7.3 mW mm^−2^ at 980 nm wavelength.^[^ ^39^, ^40^ ^]^ Using this power limitation and the unique MRI scanner environment with a strong ***B*_0_** = 7 T, a basic scaling analysis can be realized, as given in Note S1 in the Supporting Information. Required coil dimensions for the desired medical application can be approximated using these calculations. For example, this analysis estimates a maximum torque value of 106 μNm for a 2 × 2 × 2 mm^3^ sized coil by the generated 9 mA current. These calculations agree with the experimental results achieved in this work with slightly larger coils.

Most of the medical applications that use optical WPT, except for ocular applications, harvest transcutaneous light with wavelengths ranging from 770 to 850 nm (called the near‐infrared window with the deepest tissue penetration depths of several centimeters up to 5 cm).^[^ ^41^ ^]^ High optical power loss is inevitable even at these wavelengths, i.e., 30 dB loss through 3.4 cm thick bovine tissue with an 808 nm laser.^[^ ^42^ ^]^ In our case, if 980 nm laser is used with a regulation limited power intensity of 7.3 mW mm^−2^, the density would drop to 7.3 µW mm^−2^ at 2.2 cm tissue penetration depth. With the size of the solar cell used in this work, this translates into a power value of 35 µW; so, future implantable wireless sensors/communication might be possible with ultralow‐power integrated circuits.^[^ ^43^ ^]^ However, this loss cannot be pronounced for the actuators of this work since they are planned to operate on the surface of a patient inside the MRI scanner.

One limitation of the presented actuator is that its photogenerated current direction cannot be reversed to generate forces or torques in reverse directions inside the MRI scanner because of its unalterable ***B*_0_** direction. Even though, a second solar cell in reverse direction can be added to the module to gain this capability, it could cause optical power routing problems. Instead, a small‐sized application specific integrated circuit (ASIC) can be designed for this purpose in the future. Optically programmable and powered ultralow‐power ASIC can switch the connection directions between the solar cell and coil to achieve bidirectional motions. A single ASIC can be even programmed to direct power coming from a single solar cell to multiple coils to orchestrate desired motions. This would also help resolve situations where a specific unit cannot receive light to generate a desired actuation. With programmable coil selection and current direction, if a specific actuator cannot be exposed to light, the module that is orthogonal or opposite to it can be used in the reverse direction to achieve an equivalent actuation.

In this work, we developed an MRI‐compatible modular actuator and then integrated multiple of them to have actuation demonstrations that can be used in future iMRI applications. Compared to designing a specific MRI‐compatible robotic system, integrating standard modules to build functionally more complex structures is a more powerful concept and has a much broader use. A single module can be useful in marking the needle entry position or setting its orientation on the skin surface of a patient before being manually inserted by the operator. Two rotor motions around two different axes on the surface of the patient's body can be useful in iMRI applications for precise positioning, similar to the operation of a two‐axis pen plotter. The wireless spherical roller built with six modules can carry an MRI marker inside and position it on a patient by rolling and moving. These standard modules can be used in many different contexts to obtain different functionalities. It is expected that many other wireless active systems inside MRI scanners can be developed using the proposed actuator modules in this work.

## 4 Experimental Section

##### Fabrication of the Actuator Module

Monocrystalline silicon solar cell (Lemo‐Solar GmbH, Germany) was diced into 2 × 2.4 mm^2^ dies. Only dies that have narrow cathode electrode lines were used. Coils were wound in‐house using copper wires of 50 µm diameter with ≈450–500 number of turns. All the coils were wound in the same direction and the same ends of the wires were connected to the anode and cathode of the solar cell. Otherwise, actuators were not identical and generated reverse force and torque with respect to each other. Silver epoxy (Epoxy Technology, H20E‐PFC 10Z) was used to connect the leads of the coils to the anode and cathode of the solar cells. Connections were cured inside an oven for 2 h at 80 °C. The actuator modules were then coated with cyanoacrylate adhesives to increase its mechanical strength and to support connections between components.

##### 3D Printing

2 mm sized hollow cubic plastic block used as the center of the rotor and plastic sphere with 1.4 cm diameter and six rectangular prism cavities were 3D printed using Connex3 Objet260 (Stratasys Ltd., USA).

##### Used Light Sources

785 nm, 20 mW Laser, LDM785 (Thorlabs, USA); 808 nm, 450 mW Laser, BWF1‐808‐450‐E (B&W Tek Inc., USA); custom made power‐LED with 940 nm wavelength and 3.2 W power, LZ4 IR‐LED‐Module (LedEngin Inc., USA); custom made power‐LEDs with 850 nm wavelength and power of 5.3 W, ILH‐IO04‐85SL‐SC211 (Intelligent LED Solutions, UK); and 8 V, 20 W halogen optic lamp (OSRAM, Germany) were used.

##### Optical Fibers

Optical fiber used in beam bending experiments inside the MRI scanner was 6 m long, multimode, 125 µm diameter with 105 µm diameter silica core (Thor Labs, USA). Fibers used for rotor and sphere experiments inside the MRI scanner were 6 m long, multimode, 1035 µm diameter with 1000 µm silica core (Thor Labs, USA).

##### High‐Speed Camera Imaging

For rotor rotation rate measurements in air, the video recordings were made using a high‐speed camera (v641, Phantom Inc., USA) at 1000 fps.

##### MRI Scanner

High‐field small animal MRI scanner (BioSpec 70/30, Bruker) with 7 T field strength (***B*_0_**) was used in all experiments.

##### Image and Video Capture Inside the MRI Scanner

Images and videos of the bending of fiber, rotation of the rotor, and motion of the wireless sphere were captured through a wired camera inside the MRI scanner with the following properties: Complementary metal oxide semiconductor (CMOS) sensor‐based, MRI compatible, interference‐free video cameras, 12M (MRC Systems GmbH, Germany) with interference‐free LED light sources to enlighten the experimental region.

##### Used MR Image Sequence Protocols

Fiber bending experiments of MRI was made inside 1 × PBS solution. MR images were acquired using RARE sequence protocol, 1376 ms repetition time, 80 ms echo time, echo train length of 8, 180° flip angle, and 256 × 256 acquisition matrix and RAREst (fast spin‐echo for short echo time using slew‐rate‐optimized gradients) sequence protocol, 1135 ms repetition time, 24.6 ms echo time, echo train length of 60, 168° flip angle, and 128 × 128 acquisition matrix. MR images of the rotor were acquired inside agar gel using FLASH sequence protocol, 100 ms repetition time, 4.46 ms echo time, echo train length of 1, 15° flip angle, and 256 × 256 acquisition matrix.

##### Numerical Finite Element Analysis

COMSOL 5.1 Multiphysics numeric simulation engine with default solving parameters was used for beam bending. Geometric nonlinearities were included to accommodate large bending deformations, which resulted in more accurate results. A direct solver type, multifrontal massively parallel sparse direct solver (MUMPS), is used. Auxiliary sweep with relatively smaller force input values was employed at the beginning to improve the convergence of the solver. The maximum mesh element size was kept at 121 µm with a total of 3055 number of mesh elements.

##### Electrical Circuit Simulation Tool

HSPICE (Synopsys Inc., USA) was used for the circuit simulations.

##### Statistical Analysis

Experimental results represented the mean ± standard deviation of the measurements done with sample size (*n*) equals to 3.

## Conflict of Interest

The authors declare no conflict of interest.

## Author Contributions

S.M. and M.S. conceived and coordinated the project. S.M. manufactured the devices, extracted their circuit model parameters, did HSPICE simulations, prepared optical setups, and developed analytic models for beam bending and rotor operation. S.M. and O.Y. performed experiments outside the MRI scanner, analyzed results, and wrote the paper. S.M. and O.E. performed experiments inside the MRI scanner and analyzed results. O.E. performed COMSOL simulations. All authors participated in paper preparation and editing.

## Data Availability

All data acquired or analyzed during this study are included in the published article and its Supporting Information and they are available from the corresponding author on reasonable request.

## Code Availability

This study did not use any custom code that is central to the conclusions.

## Supporting information

Supporting Information

Supplemental Movie 1

Supplemental Movie 2

Supplemental Movie 3

Supplemental Movie 4

Supplemental Movie 5

Supplemental Movie 6

Supplemental Movie 7
