# Supplementary material for: Magnetic Resonance Imaging‐Compatible Optically Powered Miniature Wireless Modular Lorentz Force Actuators
Source: Adv Sci (Weinh). 2020 Dec 4;8(2):2002948. doi: 10.1002/advs.202002948 (PMC7816712; doi:10.1002/advs.202002948)
Supplement: Supplementary file 1 — Supporting Information [file ADVS-8-2002948-s001.pdf]

© 2020 Wiley-VCH GmbH

ADVANCED SCIENCE
Open Access

Supporting Information

for *Adv. Sci.,* DOI: 10.1002/advs.202002948

Magnetic Resonance Imaging-Compatible Optically Powered Miniature Wireless Modular Lorentz Force Actuators

Senol Mutlu, Oncay Yasa, Onder Erin, and Metin Sitti*

SUPPLEMENTARY INFORMATION

Magnetic Resonance Imaging-Compatible Optically Powered Miniature Wireless Modular Lorentz Force Actuators

Senol Mutlu[1,2], Oncay Yasa[1], Onder Erin[1,5], and Metin Sitti[1,3,4,*]

[1] Physical Intelligence Department, Max Planck Institute for Intelligent Systems, Stuttgart, Germany

[2] Department of Electrical and Electronics Engineering, Bogazici University, Istanbul, Turkey

[3] School of Medicine and School of Engineering, Koc University, Istanbul, Turkey

[4] Institute for Biomedical Engineering, ETH Zurich, Zurich, Switzerland

[5] Carnegie Mellon University, Mechanical Engineering Department, Pittsburgh, PA, USA

* Correspondence to: sitti@is.mpg.de

Supplementary Note S1. Scaling analysis of the Lorentz force actuator with coil size

In order to find the size of a coil for the smallest possible footprint to generate the required force and torque values that are required in magnetic resonance imaging (MRI) applications, a scaling analysis can be done for a wound coil with the parameters given in **Figure S1**. In this figure, windings start at a diameter of D_{inner} and continue along with the thickness of the coil, t_{coil}, forming an area of A. The diameter of the insulated wire is given as D_{wire}.

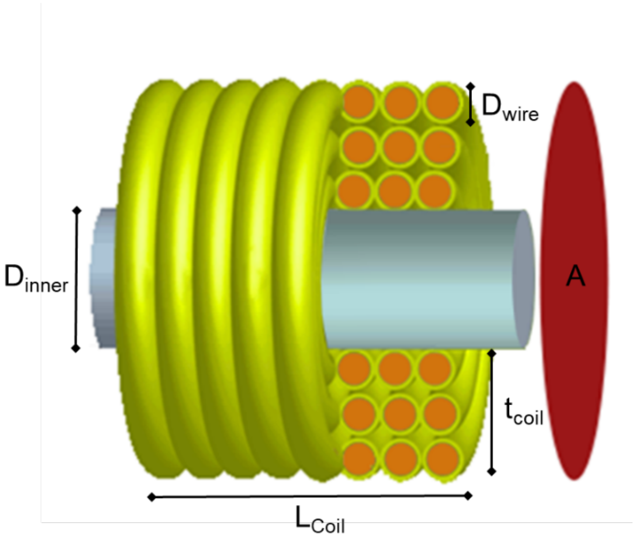

Figure S1. Depiction of a wound coil with its parameters

For a given coil size, the maximum Lorentz force that is possible to generate can be calculated as:

$$Force_{max} = BINL_{coil} \quad (1)$$

Similarly, the maximum Lorentz torque that can be generated is:

$$Torque_{max} = BINA \quad (2)$$

where N is the number of turns of the coil, I is the current through the coil and B is the uniform magnetic field magnitude.

The scaling analysis results are shown in **Table S1** as the size of the coil is made smaller so that it can be easily used in interventional MRI (iMRI) applications inside MRI scanner with $\boldsymbol{B_0}$ = 7 Tesla. The analysis assumes an insulated copper wire diameter of 50 µm for mm sized coils and 20 µm for sizes below 250 µm. The diameter of the winding wire is scaled this way so that the coil can be manufactured easily and cost-effectively. Current through the coil scales down as the area of the coil since the area of the solar cell is also matched with the coil size. A fixed continuous laser light intensity of 7.3 mW/mm^2 is used in the analysis since this is the maximum permissible exposure limit of skin under continuous exposure to 980 nm laser light by regulations[1, 2]. Instead of continuous operations if shorter durations are used, higher intensities can be used. For example, 5

s exposure usage of the same laser allows 11.9 mW/mm^2 laser power intensity. Mono-crystalline silicon solar cells have maximum sensitivity around this wavelength, hence, an optical-to-electrical power conversion efficiency of 20% and cell potential of 0.65 V is assumed in calculating the generated currents. As the table shows, a decrease in the size of the coil reduces the applicable force and torque values rapidly. This also means that by choosing a slightly larger coil size, the required force and torque values can be achieved. Depending on the applications, such as precise positioning on the skin surface of a patient, these actuators could help miniaturization efforts in iMRI.

Table S1. Scaling of the coil and the generated maximum force and torque values

Size of the coil (mm^3)	D_{inner} (mm)	D_{wire} (µm)	N (Number of turns)	Laser intensity (for 980 nm) safety limit for skin (mW/mm^2)	Current (mA)	B (tesla)	Max force (mN)	Max torque (µN.m)
4×4×4	1	50	1800	7.3	36	7	1811	7245
2×2×2	0.5	50	420	7.3	9	7	53	106
1×1×1	0.25	50	113	7.3	2.25	7	1.78	1.78
0.5×0.5×0.5	0.125	20	176	7.3	0.56	7	0.35	0.17
0.25×0.25×0.25	0.06	20	45	7.3	0.14	7	0.011	0.0028
0.15×0.15×0.15	0.03	20	17	7.3	0.051	7	0.0009	0.00014

Supplementary Note S2. Analytical approximation to large beam bending

A simple analytical approximation is developed to estimate the generated torque and beam bending angles with the current flowing through a coil inside a uniform magnetic field. The beam profile is assumed to bend symmetrically in a circular arc shape. With this simplification the bending angle can now be related to the radius of curvature, *R*, as:

$$\alpha = \frac{L}{R} \tag{3}$$

where *L* is the length and *α* is the bending angle of the beam, respectively, as depicted at the inset of **Fig. 3a**. Bending of a beam due to an externally applied moment or torque, *M*, has the following relationship with the radius of curvature[3]:

$$\mathrm{M} = \frac{Ei}{R} \tag{4}$$

where *E* is the Young's Modulus of the beam and *i* is its area moment of inertia. For a cylindrical beam bending around the axis of interest, *i* has the following expression:

$$i = \frac{\pi r^4}{4} \tag{5}$$

where *r* is the radius of the cylindrical beam. The following relationship can be derived for the generated torque magnitude using vector cross product identity:

$$\tau_{Lorentz} = INBA \sin(90 - \alpha) \tag{6}$$

where *N* is the number of turns of the coil, *I* the current through the coil, *B* the uniform magnetic field magnitude, and *A* the area of the coil. In this actuation example, an optical fiber is used both as bending cantilever beam and as an optical power guiding medium to the solar cell of the module. This way, the light power to the solar cell, hence the coil current, stays almost constant as the beam bends. Then, the following expression can be derived from equations (3), (4), (5), and (6) to find the overall expression for the bending angle.

$$\frac{\alpha}{\cos\alpha} = \frac{4BINAL}{E\pi r^4} \tag{7}$$

This analytical relationship is used to estimate the fiber bending inside the MRI scanner with $\boldsymbol{B_0}$ = 7 Tesla. As explained in the main text, this analysis is close to numerical simulations with finite element analysis and experimental results.

Supplementary Note S3. Analysis of the rotor rotation with the associated back EMF torques

To simplify the complex analysis of a 4-module rotor operation inside uniform magnetic field of $\boldsymbol{B_0}$, it is assumed that the rays of the light source are always straight and approach the rotor from one side covering its full diameter. This means that only two modules can generate electrical currents optically, namely module 1 and module 4 as depicted in **Fig. 5c** of the main text. Furthermore, the analysis is developed only for the rotation degree, θ, changing from 0° to 90° because the system is symmetric and the analysis in the rest of the quadrants are equivalent to this one with each module's identities switched.

The current flowing through module 1 generated by its solar cell results in the following expression since the light beam and the surface of its solar cell have time-varying angle between them as θ, increases from 0° to 90°:

$$I_{coil1} = I_{coilmax} \cos \omega t \tag{8}$$

where ω is the steady rotation rate of the rotor in radian/s, $I_{coilmax}$ is the maximum current passing through the coil generated by the solar cell when this solar cell is closest to the light source, *i.e.* when θ is equal to 0°. The light-powered module 1 makes 90° - θ angle with the magnetic field direction. Hence, the generated torque by this module is equal to:

$$\tau_{generated1} = I_{coil1} B_0 N A \cos \omega t \tag{9}$$

From equations (8) and (9), the generated torque expression of this module becomes:

$$\tau_{generated1} = I_{coilmax} B_0 N A \cos^2 \omega t \tag{10}$$

Using a similar derivation approach for module 4 and taking its orientation dependence on the rotation angle, it generates torque value as:

$$\tau_{generated2} = I_{coilmax} B_0 N A \sin^2 \omega t \tag{11}$$

Then, the rotor's total generated torque value can be calculated from equations (10) and (11) as:

$$\tau_{total\_generated} = I_{coilmax} B_0 N A \tag{12}$$

Since all the modules rotate steadily with the rotation rate of ω, coils of every module generate back EMF with different polarities so that all of them oppose rotation, including the modules that generate motion torques. Each module has a specific dependence on the rotation angle, θ and all of them are orthogonal to each other. Hence, each one is investigated separately. The generated back EMF torques of the modules are labeled as in **Fig. 5c** of the main text. Module 1, which also generates rotation torque, generates back EMF value with the following expression:

$$V_{backemf1} = -\mathrm{N}\frac{d\Phi}{dt} = B_0 N A \omega \cos \omega t \tag{13}$$

This voltage generates a current in the coil depending on the impedance that it sees. In this case, it is the sum of the impedance of the coil and impedance of the solar cell, which can be expressed as

Z. Please note that this kind of analysis is possible because of the superposition theorem of electrical circuits. This theorem helps to find the response of a circuit element in a circuit that has more than one independent source. This is achieved by activating each independent source alone, deactivating the rest of the independent sources, but keeping their impedances, and then summing the responses due to all independent sources[4]. Then, the current that is generated through the coil due to this back EMF is:

$$I_{backemf1} = \frac{B_0 NA\omega \cos \omega t}{Z} \tag{14}$$

This, in turn, generates a back EMF torque with the following expression:

$$\tau_{backemf1} = \frac{B_0^2 N^2 A^2 \omega \cos^2 \omega t}{Z} \tag{15}$$

The generated back EMF of the first module reverse biases the solar cell. As characterized in **Fig. 2d**, the reverse direction of the solar cell passes a very small amount of current even for tens of negative voltages applied. As a result, very small back current can flow due to this back EMF, and torque of this module is very small. Z value for this reverse-biased solar cell case can be labeled as $Z_{reverse}$, which is very high. The fourth module is similar to this module. It generates back EMF current that reverse-biases the solar cell and has similar expression but with sine function instead of cosine function since it is orthogonal to the module 1.

The second module generates back EMF current in the forward bias direction of the solar cell as marked in **Fig. 5c**. Hence, it can generate a large amount of current and torque value. Based on its rotation orientation with respect to the rotation angle, current and torque expressions contain sine function instead of cosine, and Z value is for the forward-biased solar cell case. This impedance can be labeled as $Z_{forward}$. It is approximately equal to the sum of the coil impedance and the on-resistance value of the solar cell. Similarly, module 3 generates back EMF current that forward biases the solar cell. Its expressions contain cosine function based on its orientation. Then, the total back EMF torque expression becomes:

$$\tau_{backemf} = \frac{B_0^2 N^2 A^2 \omega \cos \omega t \cos \omega t}{Z_{reverse}} + \frac{B_0^2 N^2 A^2 \omega \sin \omega t \sin \omega t}{Z_{forward}} + \frac{B_0^2 N^2 A^2 \omega \cos \omega t \cos \omega t}{Z_{forward}} + \frac{B_0^2 N^2 A^2 \omega \sin \omega t \sin \omega t}{Z_{reverse}} \tag{16}$$

This expression reduces to:

$$\tau_{backemf} = B_0^2 N^2 A^2 \omega \left(\frac{1}{Z_{forward}} + \frac{1}{Z_{reverse}}\right) \tag{17}$$

Since $Z_{reverse}$ is much bigger than $Z_{forward}$, it further reduces to:

$$\tau_{backemf} = \frac{B_0^2 N^2 A^2 \omega}{Z_{forward}} \tag{18}$$

Finally, for a steady rotation rate of ω, the total generated torque value in (12) must be equal to the total opposition torque in equation (18) generated by back EMFs. From this equality, the following expression can be found for ω:

$$\omega = \frac{I_{coilmax} Z_{forward}}{B_0 N A} \quad (19)$$

From this analytical result, we expect the rotation rate to increase linearly with the current flowing through the coil hence the light intensity as long as saturation does not happen in the solar cell. It should also increase with the reciprocal of the magnitude of the magnetic field.

This analysis can be solved numerically with the help of the HSPICE computer-aided design (CAD) tool. Equivalent circuit model, shown in **Fig. 1b**, is constructed for each module. Photogenerated current source value for module 1 is entered in the form of equation (8) to HSPICE for a given ω value. Similarly, it is done for module 4. The program calculates the generated voltages and currents in all of the four modules. Expressions of equation (10), (11), and (16) are calculated by HSPICE and transient results are plotted on a graph. If the generated torque value is higher than the total back EMF torque value, the simulation is iterated with a higher ω value. If the simulation results show a higher back EMF torque value than the generated torque, ω value is reduced in the next iteration. The simulations are repeated until ω value is found to make torque values equal. An example of an HSPICE simulation is shown in **Figure S2**. For 16 mA photogenerated current value, which generates around 15.8 mA coil current, with $\boldsymbol{B_0}$ = 7 Tesla and ω value of 37.7 radian/s (meaning 360 rpm), the resulting generated and back EMF torques are plotted for θ rotation angle from 0° to 90°. The torque values have almost the same magnitude of 454 µNm but in opposite directions, meaning that this rotation can be sustained. In this figure, two components of the generation torques and four components of the back EMF torques are also plotted individually. As explained earlier, back EMF1 and back EMF4 are negligible since they reverse bias the solar cells. Back EMF2 and back EMF3 generate high opposing torques with peaks at different time instances since they are oriented orthogonal to each other. Rotor experiments inside the MRI scanner result in 239 rpm. This much disagreement is expected since these analytical and numerical solutions do not take friction, the asymmetric center of mass, gravity effects, and small laser spot size into account. Even though simulation results overestimate rpm values, it gives an intuition about the working mechanism of this type of rotor. It predicts correctly how rpm and torque values change with the change in the current and magnetic field.

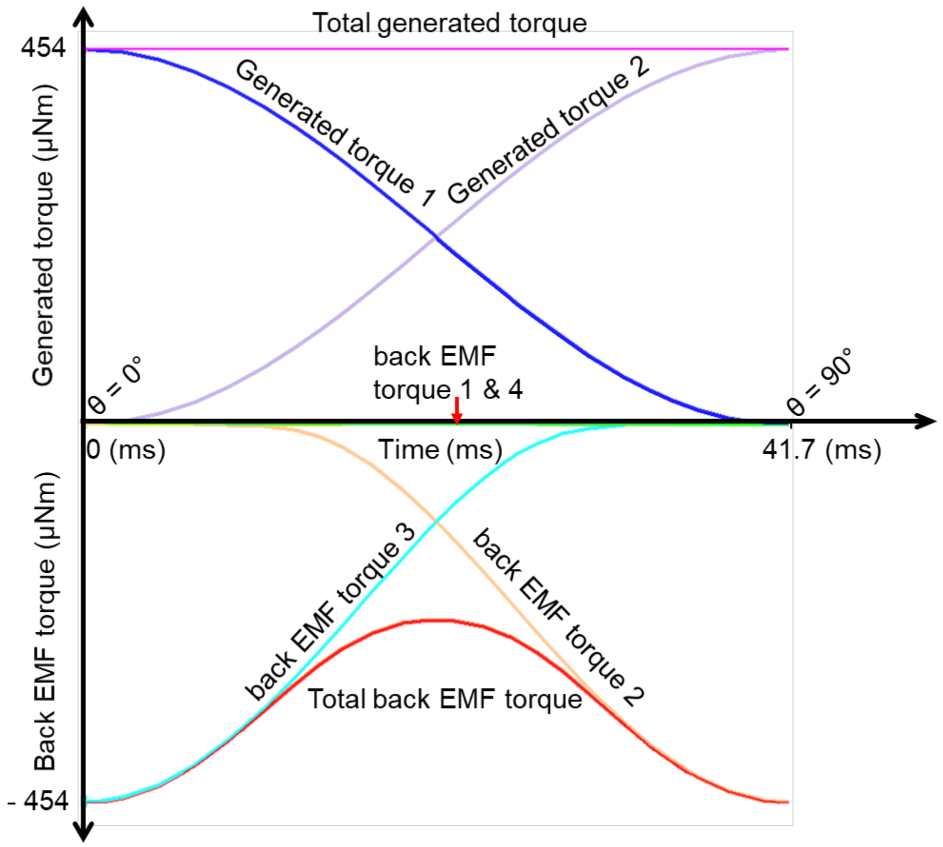

Figure S2. HSPICE simulation results showing total generated torques and total back EMF torques as well as individual contributions of each module for 16 mA photogenerated current, uniform magnetic field of $\boldsymbol{B_0}$ = 7 Tesla, and 360 rpm rotation rate.

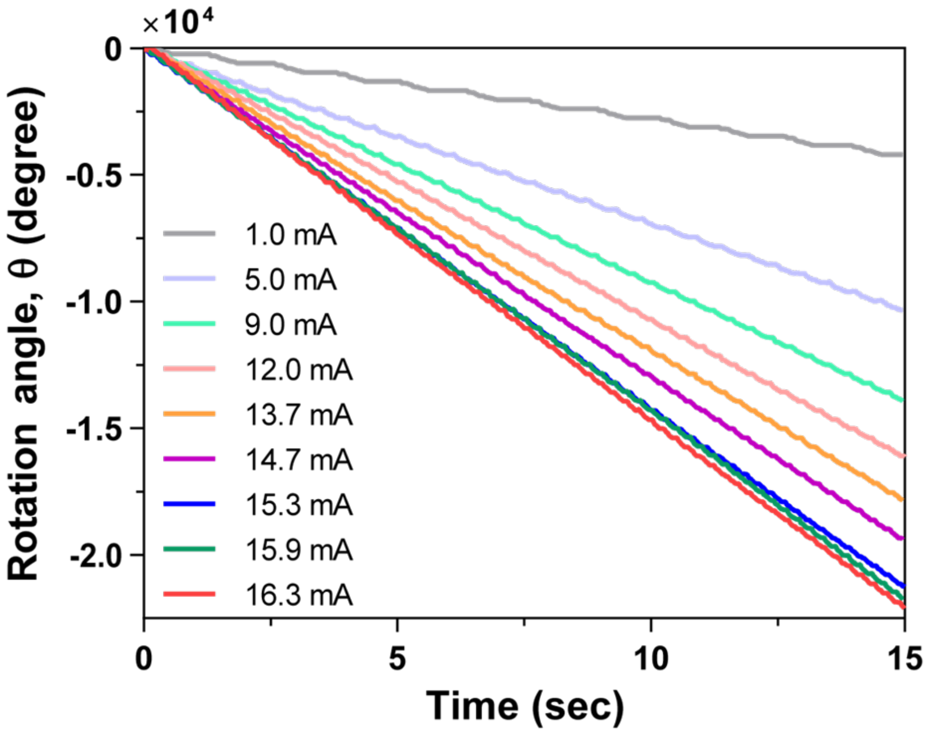

Figure S3. Detailed experimental results of the change in the rotation angle of the rotor in the air inside the preclinical MRI scanner ($\boldsymbol{B_0}$ = 7 Tesla) with time for different optical power values resulting in different current values through the coils. Instantaneous rotation rate changes with time because of the asymmetry of the rotor, the position of the center of the mass, gravity effects, friction, and small-sized light spots.

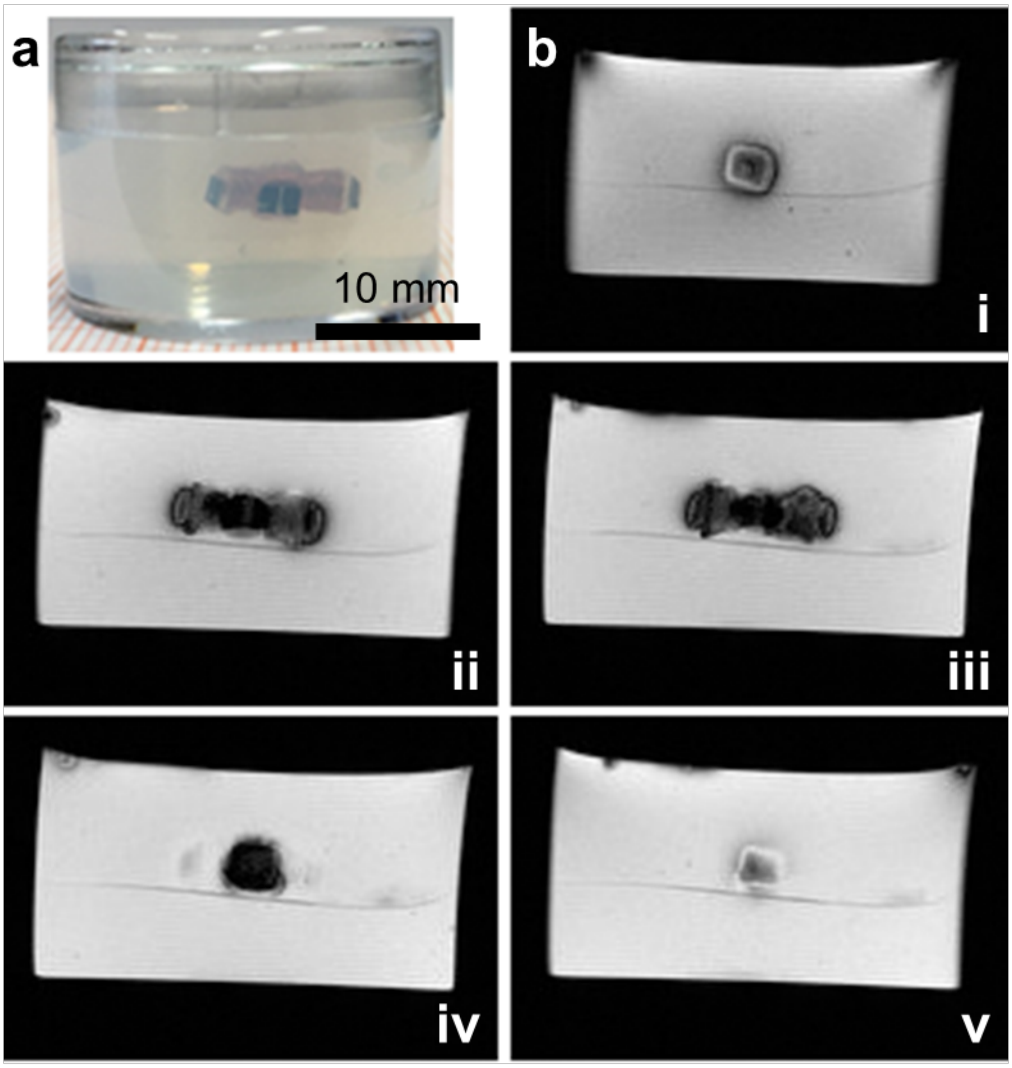

Figure S4. a. Optical image of the rotor inside the agar gel. **b.** Axial MR image slices of the same rotor inside preclinical MRI scanner (B_0 = 7 Tesla) showing no image artifacts when it is not operated. FLASH protocol of 100 ms repetition time, 4.46 ms echo time, echo train length of 1, 15° flip angle, and 256×256 acquisition matrix is executed.

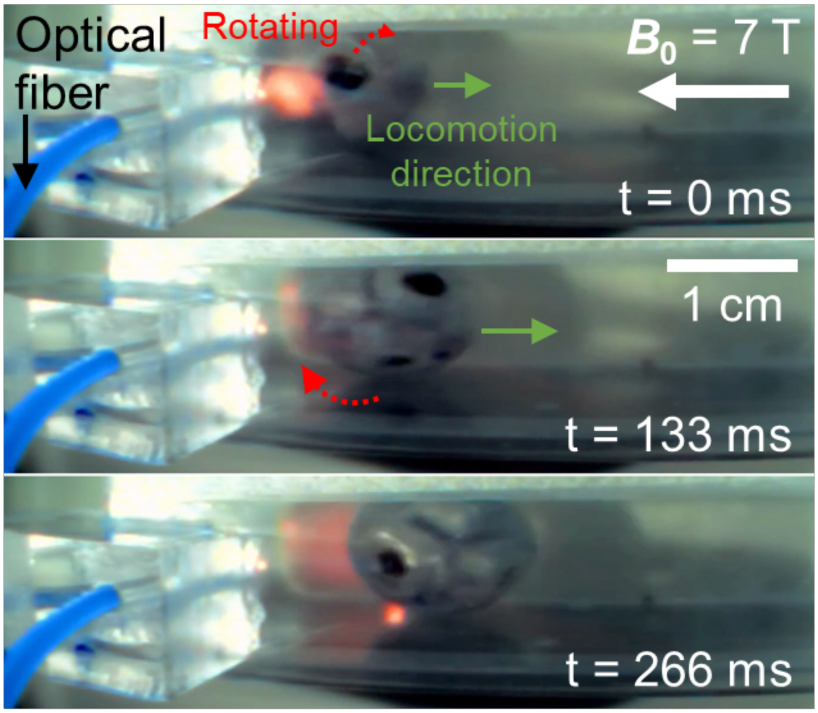

Figure S5. Rotation and forward motion of the sphere inside the preclinical MRI scanner ($\boldsymbol{B_0}$ = 7 Tesla) inside a plastic container upon turning on IR light through the optical fibers

Supplementary Movies

Movie S1. Two free modules actuated freely on a magnet [Real-time actuation of two free wireless optically driven miniature Lorentz force modules are demonstrated on the surface of a 0.1 T permanent magnet by a spot of 808 nm laser beam light].

Movie S2. Sample real-time bending actuation results of cantilever beams inside the MRI scanner [The actuator module is attached to the tip of an optical fiber beam (Length of 7 mm and diameter of 105 µm silica core). Its bending is tested inside the MRI scanner's uniform field, $\boldsymbol{B_0}$ = 7 Tesla. The laser power is turned on and off and its intensity is adjusted to generate the desired current values in the range of 0 to 12.8 mA in the actuator coil. Sudden motion jumps in the movie are results of these adjustments.]

Movie S3. Real-time rotation of rotor built using four modules in the air over a permanent magnet [An example of the rotation of the rotor in the air with rotations rates in the order of 1000 rpm on a 0.15 Tesla magnet by an 808 nm laser from a distance of 12 cm.]

Movie S4. Real-time rotation of rotor inside silicone oil with different viscosities and magnetic field magnitudes [Rotors are inside silicone oils with viscosities of 100 cSt, 1000 cSt, and 10000 cSt. The rotation rates are measured and mentioned in the movie over 0.17 Tesla and 0.32 Tesla magnets using a 20 W halogen lamp from a distance of 2 cm, giving approximately an optical power density of 12 mW/mm^2.]

Movie S5. Real-time rotor rotations inside the MRI scanner ($\boldsymbol{B_0}$ = 7 Tesla) for different optically generated coil currents [Rotors is inside a plastic box and two optical fibers are positioned around it with 45° between them. Rotation rates change as the laser intensity through one fiber is increased.]

Movie S6. The real-time spinning of a sphere built with six modules on a magnet and its changing direction [The sphere starts to spin at rotation rates of around 1800 rpm by itself as soon as the infra-red (IR) LED light is turned on. A 5.3 W power-LED of 850 nm wavelength is used on the side of 0.25 Tesla permanent magnet.]

Movie S7. Real-time rolling of the sphere inside the MRI scanner ($\boldsymbol{B_0}$ = 7 Tesla) [The sphere is positioned inside a plastic box and two optical fibers are positioned around it with 45° between them. As two fibers send optical power, generated torques on the modules roll the sphere and move it forward along the $\boldsymbol{B_0}$ direction of the MRI scanner.]

References

1. Protection ICoN-IR. ICNIRP Guidelines on Limits of Exposure to Laser Radiation of Wavelengths between 180 nm and 1,000 µm. *Health physics* **105**, 271-295 (2013).
2. Yun SH, Kwok SJ. Light in diagnosis, therapy and surgery. *Nature biomedical engineering* **1**, 1-16 (2017).
3. Timoshenko S, Goodier J. Theory of Elasticity, 3rd Edn McGraw-Hill. *New York*, (1970).
4. Alexander CK, Sadiku MN. *Fundamentals of electric circuits*. McGraw-Hill Education (2000).
